# Different audio spatial metric representation around the body

**DOI:** 10.1038/s41598-018-27370-9

**Published:** 2018-06-20

**Authors:** Elena Aggius-Vella, Claudio Campus, Monica Gori

**Affiliations:** U-VIP: Unit for Visually Impaired people, Center for Human Technologies, Istituto Italiano di Tecnologia, Genoa, Italy

## Abstract

Vision seems to have a pivotal role in developing spatial cognition. A recent approach, based on sensory calibration, has highlighted the role of vision in calibrating hearing in spatial tasks. It was shown that blind individuals have specific impairments during audio spatial bisection tasks. Vision is available only in the frontal space, leading to a “natural” blindness in the back. If vision is important for audio space calibration, then the auditory frontal space should be better represented than the back auditory space. In this study, we investigated this point by comparing frontal and back audio spatial metric representations. We measured precision in the spatial bisection task, for which vision seems to be fundamental to calibrate audition, in twenty-three sighted subjects. Two control tasks, a minimum audible angle and a temporal bisection were employed in order to evaluate auditory precision in the different regions considered. While no differences were observed between frontal and back space in the minimum audible angle (MAA) and temporal bisection task, a significant difference was found in the spatial bisection task, where subjects performed better in the frontal space. Our results are in agreement with the idea that vision is important in developing auditory spatial metric representation in sighted individuals.

## Introduction

To date, most of the experiments testing auditory spatial representation have focused on the frontal space, mostly at ear level. However, several pieces of research have shown that the space around the body is not coded as unitary dimension, but it is split into multiple regions in which stimuli are differently perceived. The major divisions are between far and near space^1–4^, space around different body regions^5–7^, and left and right, as shown in neglect patients^8^. A less investigated space is the back zone. This zone is particularly interesting, as vision and action are not easily available there. The back space might be differently processed from the frontal space, as suggested by studies on neglect^9–11^ and on multisensory integration^12,13^, where a facilitatory effect of audio tactile stimuli interactions was found in the rear space^12^. An interesting experiment on temporal order judgment^14^ showed that the spatiotemporal representation of non-visual stimuli in front and rear space is different. In this experiment, stimuli were delivered on crossed and uncrossed hands in two different spaces: frontal and rear space. Results showed a significantly better performance in the crossed posture, when the hands were placed in the back space, than in the frontal space. This finding is particularly interesting, as previous experiments on blind and sighted people showed that congenitally blind individuals do not demonstrate any impairment in tactile temporal order judgment tasks (TOJs) as a result of crossing their hands^15,16^. It is possible to speculate, therefore, that underlying mechanisms similar to those adopted by blind people, might also be adopted by sighted people in space where vision is not available^14^. Interesting, another study^17^ showed that, the impairment during crossed posture, is still present around the legs (ankles). The study revealed that audio, visual information, and their integration, differently affect the localization of tactile stimuli. This suggests that mechanisms, controlling the alignment between somatotopic and external reference frames, include spatial features conveyed by the auditory and visual modalities. To date, it is not clear how vision differently influences the perception of stimuli in spaces around us. If vision is important in spatial representation, then we might expect visual and non-visual spaces to be differently represented. On one hand, the sensory compensation hypothesis^18,19^ states that the lack of a sensory ability (e.g. vision), leads to an improved ability of non-visual senses. Studies on blind subjects support the idea that this enhanced auditory ability is due to cross-modal plasticity^20,21^. The visual cortex is highly plastic; this is more evident in young animals, but it is still present in adulthood^22^. This plasticity allows the visual cortex in congenitally blind people to become colonized by other sensory systems (i.e. auditory and somatosensory)^23,24^. Few days of binocular deprivation is sufficient for the primary visual cortex to be colonized by touch^25^. There is also psychophysical evidence that the congenitally blind have enhanced tactile discrimination^26^, auditory pitch discrimination^27^, sound localization^28,29^, and are able to properly form spatial topographical maps^30,31^. Spatial hearing tasks have been shown to activate the visual cortex of early blind individuals^24,31–33^, and individual localization abilities have been shown to correlate with the magnitude of visual cortical activity^31,32,34,35^. Interestingly, the enhancement is not uniform, but depends somewhat on the space considered. For example, localization of peripheral, but not central stimuli exceeds that of controls^29^, and is similar for the localization along the horizontal, but poorer for the vertical meridian^36^. This is consistent with anatomical evidence showing that the peripheral visual field has strong auditory projections^37^, possibly facilitating colonization.

**Figure 1:**
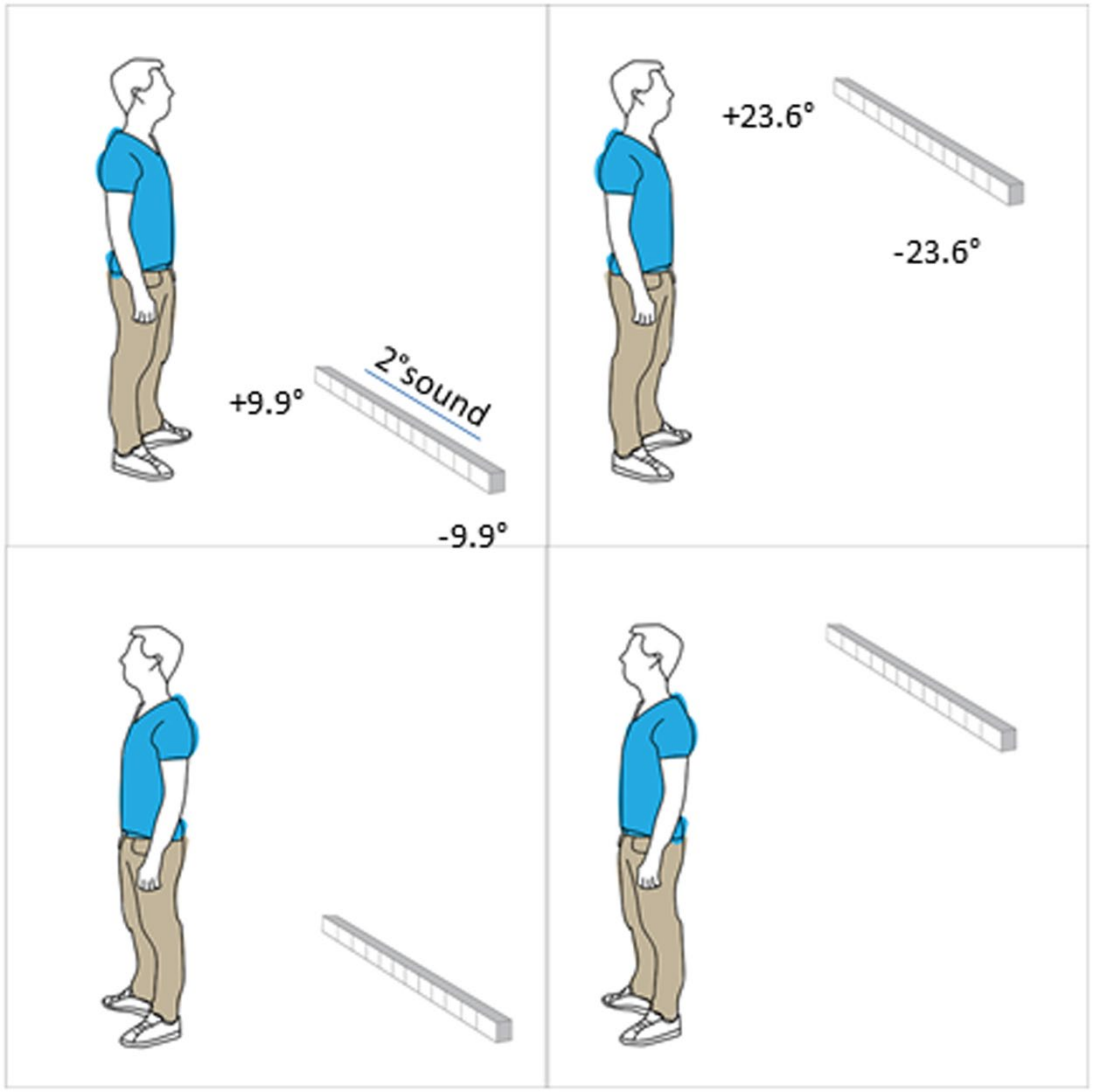
Set up and tasks. The spatial bisection task was performed at ear and foot level in both frontal and rear space. Three sounds were delivered from the three different speakers. Subjects had to judge whether the second sound was closer to the first or to the last sound. The spatial order of the three sounds was fixed in the space (they always started from the same speaker). Minimum audible angle task: the task was performed at ear and foot level in both frontal and rear space. Subjects had to judge the position of two sounds. In the frontal condition, subjects had to report which of the two sounds was further right, while in the rear condition, they had to state which of the two sounds was further left. Temporal bisection was performed at ear and foot level in both frontal and rear space. Three sounds were delivered from the same speaker with different delays between each other, and subjects had to judge which was the shortest interval between sounds.

On the other hand, the general-loss hypothesis states that vision has a pivotal role in developing spatial cognition^38–40^. Indeed, vision allows multiple stimuli to be judged simultaneously, leading to the possibility of building spatial relationships between objects displaced around us^41^. This key role of vision in spatial perception is explained well by the sensory calibration theory, which states that the more accurate sense teaches (calibrates) the others; when one calibrating modality is missing, the other modalities result impaired. Children with visual disabilities have problems in understanding the haptic or auditory perception of space^42^ and children with motor disabilities have problems in understanding the visual dimension of objects^43^. This theory shows that congenitally blind people are impaired in several spatial tasks^42,44–51,58^. The auditory spatial impairment in some tasks, such as in spatial bisection, seems to be related to the inability to build a spatial metric between sounds^44^. This phenomenon is well illustrated in a study comparing sighted blindfolded people and blind people, while performing a spatial bisection task^44^. The study showed an impairment in the congenitally blind subjects. To correctly perform the task, participants need to listen to a sequence of three sounds, located in different spaces, and identify the spatial position of the second sound with respect to the other two sounds. This task relies greatly on a topographical spatial map. The deficit was absent for other simpler tasks, such as the minimum audible angle, in which a relative comparison between the sound sources is enough to perform the task. Authors suggested that blind subjects had a preserved topological representation of space, but an impaired Euclidian representation. If vision is important in spatial representation, then we might expect the frontal zone to be differently represented. In this paper we investigated this point, testing auditory space representation around the body by administering a spatial bisection task, a temporal bisection task and a minimum audible angle (MAA) task in the frontal and back zones. We replicated the test at two body levels: the ear and the foot (Fig. 1). These conditions were adopted to ensure that possible differences in the performance of the spatial bisection task were not due to different acuity in the auditory perception (MAA) or to a different ability to comprehend the auditory metric (temporal bisection task) in different spatial positions, as well as to ensure that the effect was strictly related to just the back space and not a mere effect due to a specific part of the body. Finally, as a further control, the temporal bisection task was adopted in order to ensure that sounds coming from the four different spaces were equally perceived. Results confirm our hypotheses by showing a poorer performance in the back than in the frontal space. As expected, the difference was evident only in the bisection task and not for the minimum audible angle or for the temporal bisection task. Moreover, it was present at both body levels considered: at the ear and at the foot level. Finally, the opposite pattern was found in the MAA. This result supports the hypothesis that vision plays a key role in developing spatial metric representation that, in turn, adjusts the construction of an auditory spatial metric representation in our brain.

## Materials and Methods

Twenty-three subjects (7 females and 16males) aged 28 ± 11years (mean ± SD) took part in the experiment, and all participants confirmed they had normal hearing and no cognitive impairments. All subjects performed the spatial bisection task, the minimum audible angle and the temporal bisection task in a randomized order. Subjects were blindfolded before entering a normal experimental room (echoic), so they had no notion of the room or the speaker layout. Subjects were standing at a distance of 80 cm from the stimuli and aligned with the center of a bank of 11 speakers, spanning respectively ±23.6° of visual angle, at ear level and ±9.9° at foot level, distance between 2 near speakers was of 7 cm. The position was continuously monitored by the experimenter. We used an onset abrupt pink noise lasting 100 ms, for which both interaural time differences and interaural level differences are important; the sound was well heard from every subjects. For the spatial bisection task, three 100 ms stimuli were presented successively at 500 ms intervals between 11 speakers, the first at −23.6° (at ear level) or −9.9° (at foot level), the third at +23.6°(at ear level) or +9.9°(at foot level), based on condition, and the second at an intermediate speaker (between the first and third sound, 9 possible speakers) position determined by the QUEST adaptive algorithm^52^, which estimates the point of subjective equality after each response, and places the next trial near that estimate. Subjects reported verbally whether the second sound was closer to the first or to the last sound. Each subject performed 60 trials for each condition.

For the minimal audible angle task, two 100 ms stimuli of 500 Hz were presented successively with a 500 ms interval, one (randomly first or second) on the central speaker (0°), the other at a certain distance left or right, following the QUEST algorithm.

Each task was carried out at two elevations (ear and foot level) and in two different positions (front and back), resulting in four randomized conditions: frontal ear, frontal foot, back ear and back foot space. In order to perform the task in allocentric coordinates, the spatial order of the three sounds was always the same, independent from the position of the subjects. This means that, in the frontal condition of the bisection task, the first sounds started from the left of the subjects’ position, while in the back condition the sound started from the right (same absolute position in the space but different in relation to the body). In the MAA, in the frontal conditions the subjects reported which of the two sounds was located further right, and in the back conditions, which was further left (see Fig. 1). Each subject performed 60 trials for each condition. For the spatial bisection task, the proportion of “third” responses was calculated for each speaker distance, while for the MAA task the proportion of right or left (in accordance with condition) was calculated.

The temporal bisection task was used as a control task in order to ensure that sounds coming from the four quadrants were similarly perceived. The task is similar to the spatial bisection, except that all sounds were played on the central speaker (0^°^), and subjects reported verbally whether the middle sound was temporally closer to the first or the last (the total duration was still 1 s, but the second stimulus varied in time, following the QUEST algorithm). For all tasks, results were fitted by Gaussian error functions whose standard deviation estimated threshold. Most subjects completed all the tasks in one session, with an interval between each task and each condition.

All participants gave written informed consent before starting the test. The study was approved by the ethics committee of the local health service (Comitato etico, ASL 3, Genova) and conducted in line with the Declaration of Helsinki.

## Analysis and Results

**Figure 2:**
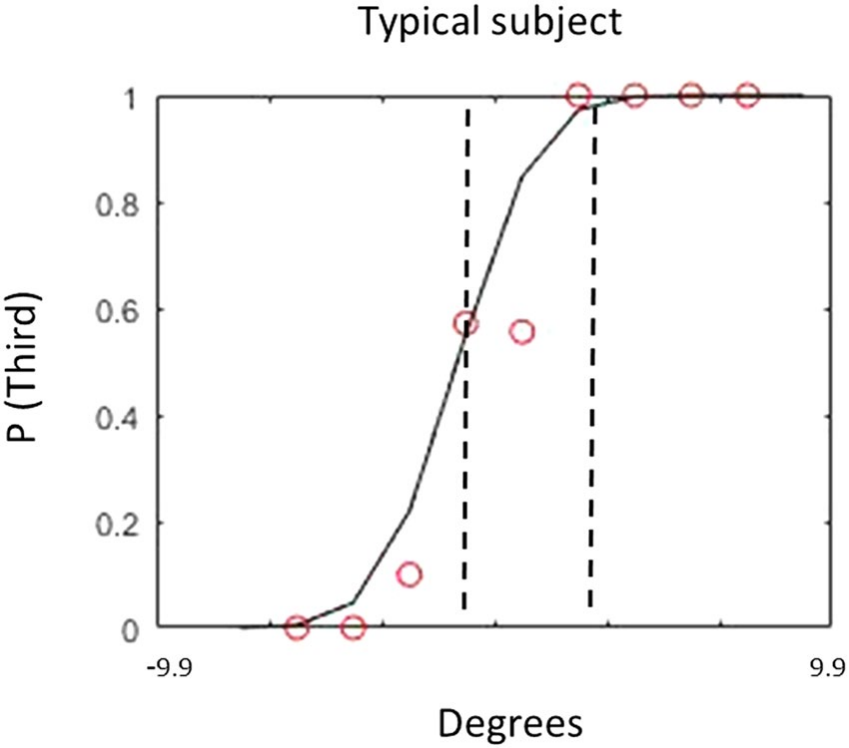
Performance of a typical subject in the spatial bisection. On the y axis is represented the probability that the second sound was closer to the third sound, while on the x axis are represented the degrees. Degrees are calculated on the base of ear distance from the sound source. As shown in the figure, from the psychometric curves, we estimated not only the perceptive threshold (PSE) but also the variability around the threshold (precision), represented by the two dashed lines represent. Specifically, we evaluated precision as the steepness of the psychometric curve fitted to the data, which is the SD of the corresponding Gaussian curve.

Twenty-three subjects participated in the experiment and performed a total of 3 tasks, namely spatial bisection, minimum audible angle (MAA) and temporal bisection. Each task was carried out at two elevations (ear and foot level) and in two different positions (front and back), resulting in four randomized conditions: frontal ear, frontal foot, back ear and back foot space (Fig. 1). For each task, each condition consisted of 60 trials, for a total of 720 trials for each subject. For each condition, a psychometric function was calculated. For each subject and condition, the space constant (σ) of the fit was taken as the estimate of threshold for all tasks (Fig. 2 for a typical subject). Space constants were converted from centimeters to angles. However, the distance of speakers from the ears was different at ear level (80 cm) and foot level (200 cm), thus corresponding to different visual angles between the first and third sound (±23.6° and ±9.9° respectively).

**Figure 3:**
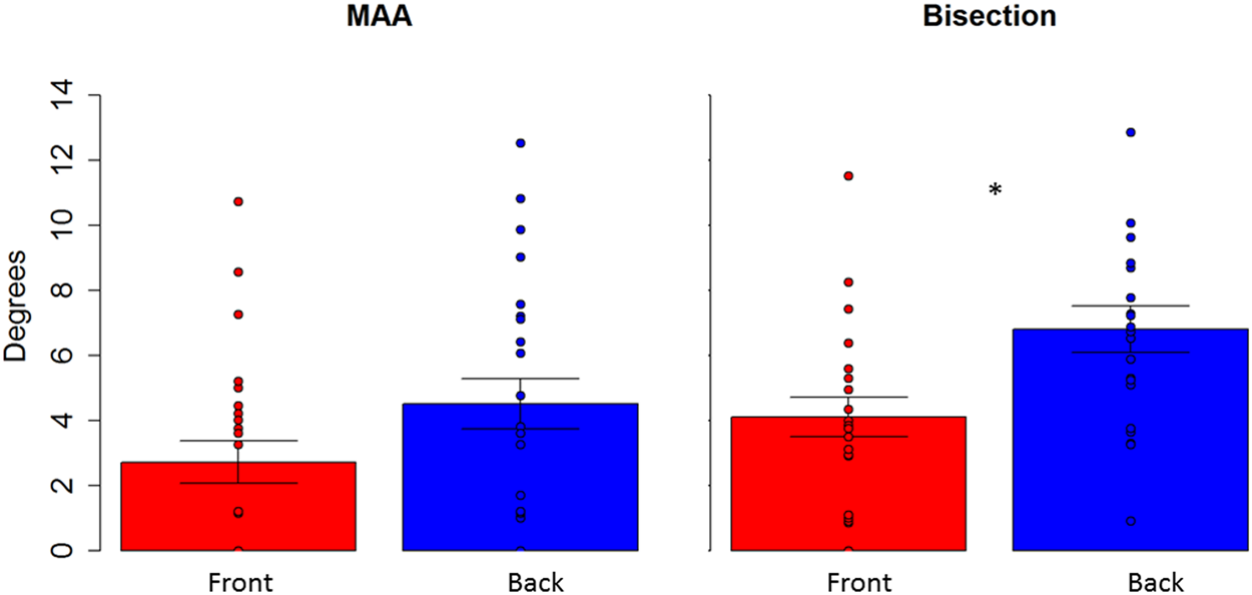
Thresholds in the spatial bisection and MAA at ear level. red bar reports threshold (deg) for frontal condition, while blue bar refers to back condition. Dots represent the performance of each subject. As can be seen, subjects were more precise in the frontal space than in the back, suggesting that vision plays an important role in calibrating spatial hearing. No difference between frontal and back was found in the MAA. ^*^Indicated p < 0.05.

**Figure 4:**
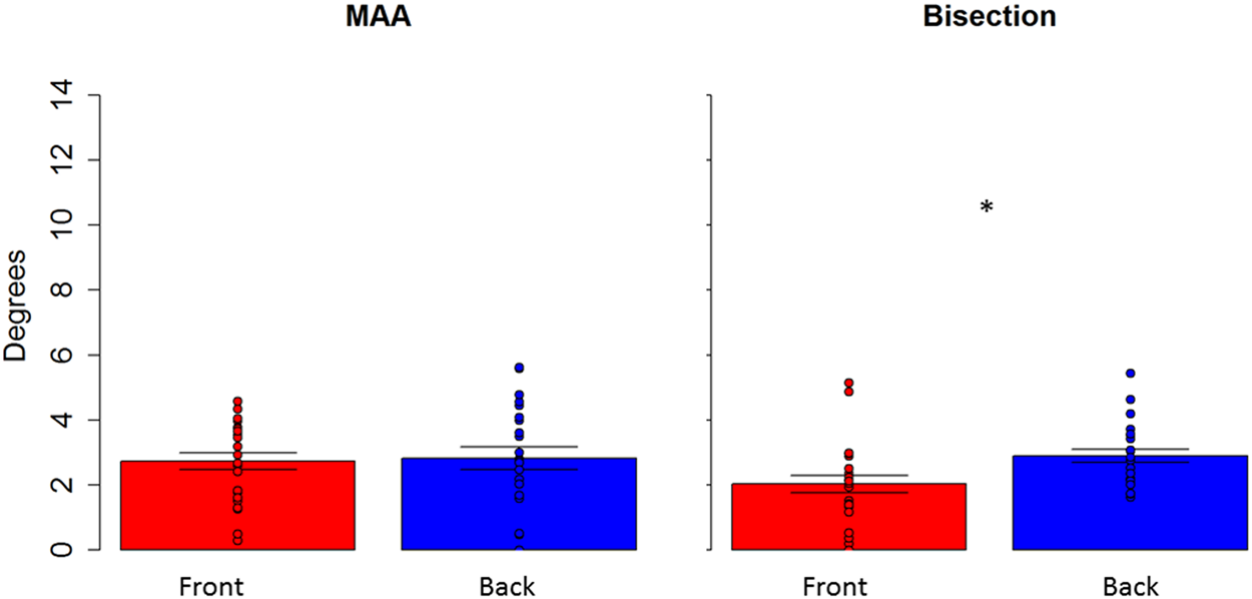
Thresholds in the spatial bisection and MAA at foot level. red bar reports threshold (deg) for frontal condition, while blue bar refers to back condition. Dots represent the performance of each subject. As can be seen, subjects were more precise in the frontal space than in the back. No difference between frontal and back was found in the MAA. ^*^Indicated p < 0.05.

**Figure 5:**
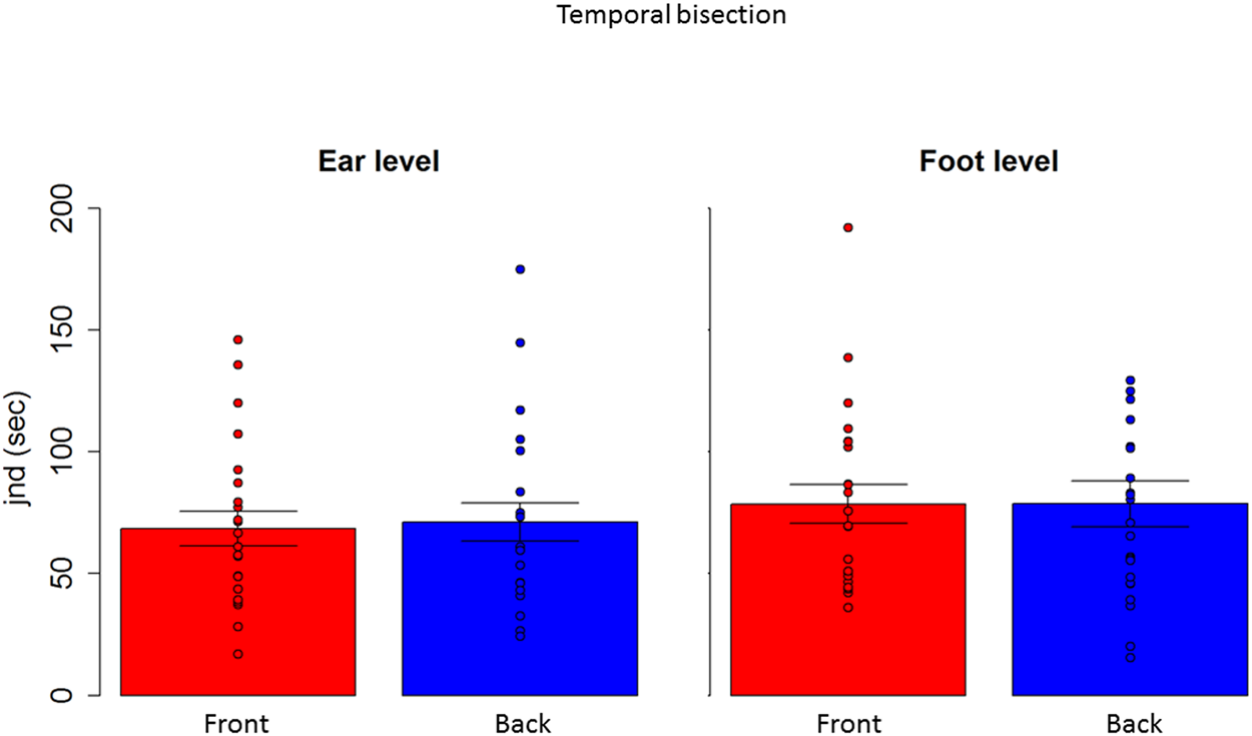
Thresholds in the temporal bisection. No difference between front (red bar) and back (blue bar) conditions were found at either ear or foot level. Dots represent the performance of each subject.

Therefore, in a first analysis, we considered each level separately and we compared front and back position with a paired two tailed t-test and we considered P < 0.05 as significant, after applying Bonferroni correction for multiple comparisons (mean and standard error are reported). A T test performed on the spatial bisection task (Fig. 3 right) at ear level showed a significantly higher precision (lower threshold) (t_(22)_ = −4.7, P < 0.01) in the frontal space (4.1 +/− 0.6), compared to the back space (6.8 +/− 0.7). The same analysis performed on the MAA (t_(22)_ = −1.9, P = 0.06), (Fig. 3 left) and on the temporal bisection tasks (t_(22)_ = −0.3 P = 0.7) showed no difference between frontal and rear space. This result is clearly shown in Fig. 3, where results from the two tasks are reported. Blue bars represent the threshold obtained for the back space, while the red bar represents the frontal space. As can be seen, no differences are reported between threshold for the front and rear space in the MAA, while a significant lower precision (higher threshold) is evident in the back space during the spatial bisection task. To be sure that the higher precision (lower threshold) in the frontal space was not an effect of higher saliency of space around the face, the same comparison was performed for the threshold obtained for the test carried out at foot level. Exactly the same pattern of results was found at foot level. As reported in Fig. 4, higher precision (lower threshold) is present when performing the spatial bisection task in the frontal space than in the back space (t_(22)_ = −2.2, P < 0.01), while no difference was observed between front and back in the MAA (t_(22)_ = −0.3, P = 0.7). Significantly, no difference between frontal and rear space was found in threshold for the temporal bisection tasks at either ear level (t_(22)_ = −0.3 P = 0.7) or foot level (t_(22)_ = −0.007, P = 1), as reported in Fig. 5, suggesting that the complexity of performing the bisection task, in the temporal domain, was similar for the 4 spaces.

**Figure 6:**
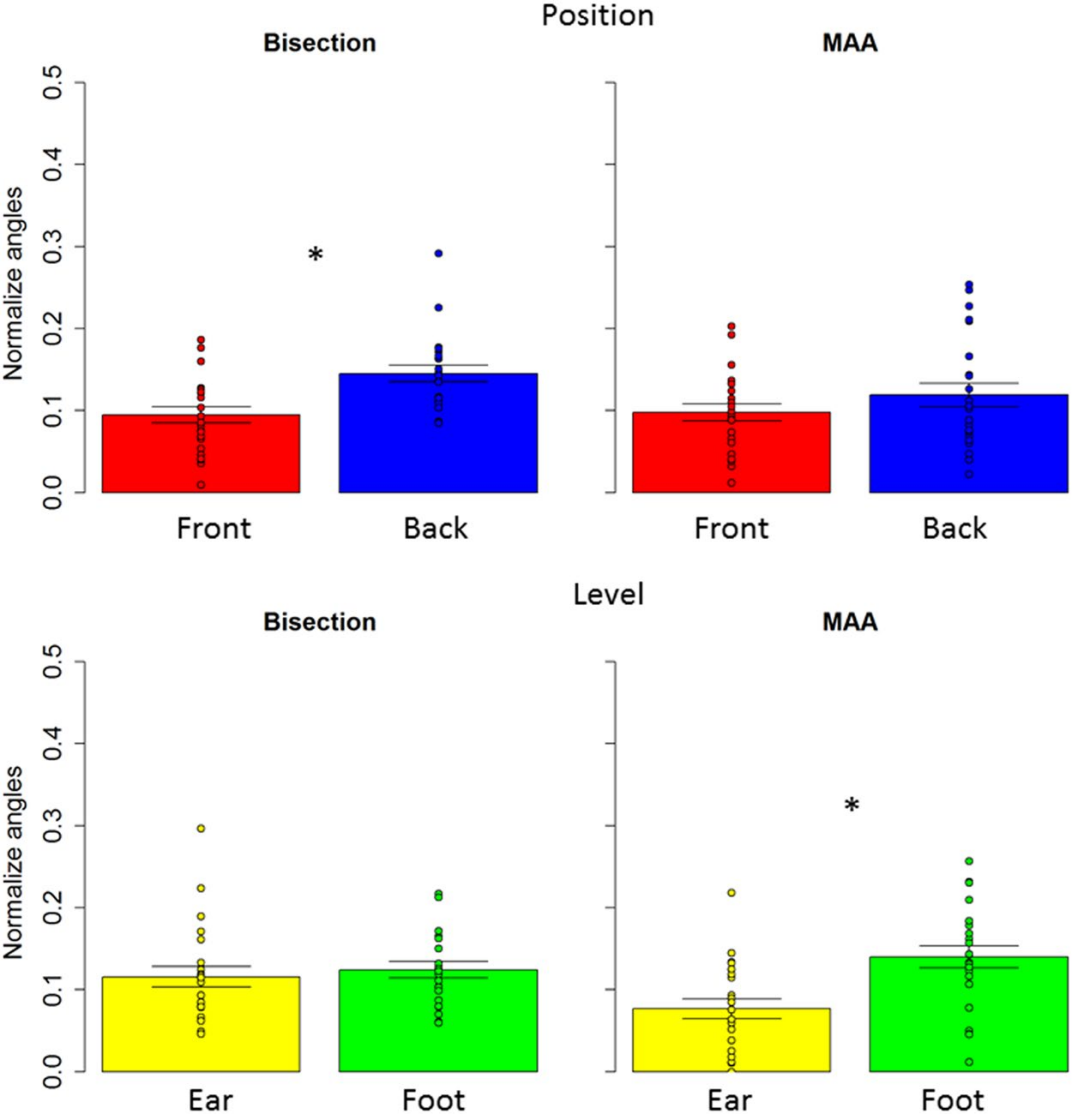
Normalized thresholds in the spatial bisection and MAA. red bar reports threshold (deg) for frontal condition, while blue bar refers to back condition. Dots represent the performance of each subject. As can be seen, in the bisection task, subjects were more precise in the frontal space than in the back, suggesting that vision plays an important role in calibrating spatial hearing, while no difference were found between ear (yellow) and foot (green) level. An opposite pattern was found in the MAA: no difference between frontal and back, while a difference was found between ear and foot level. ^*^Indicated p < 0.05.

**Figure 7:**
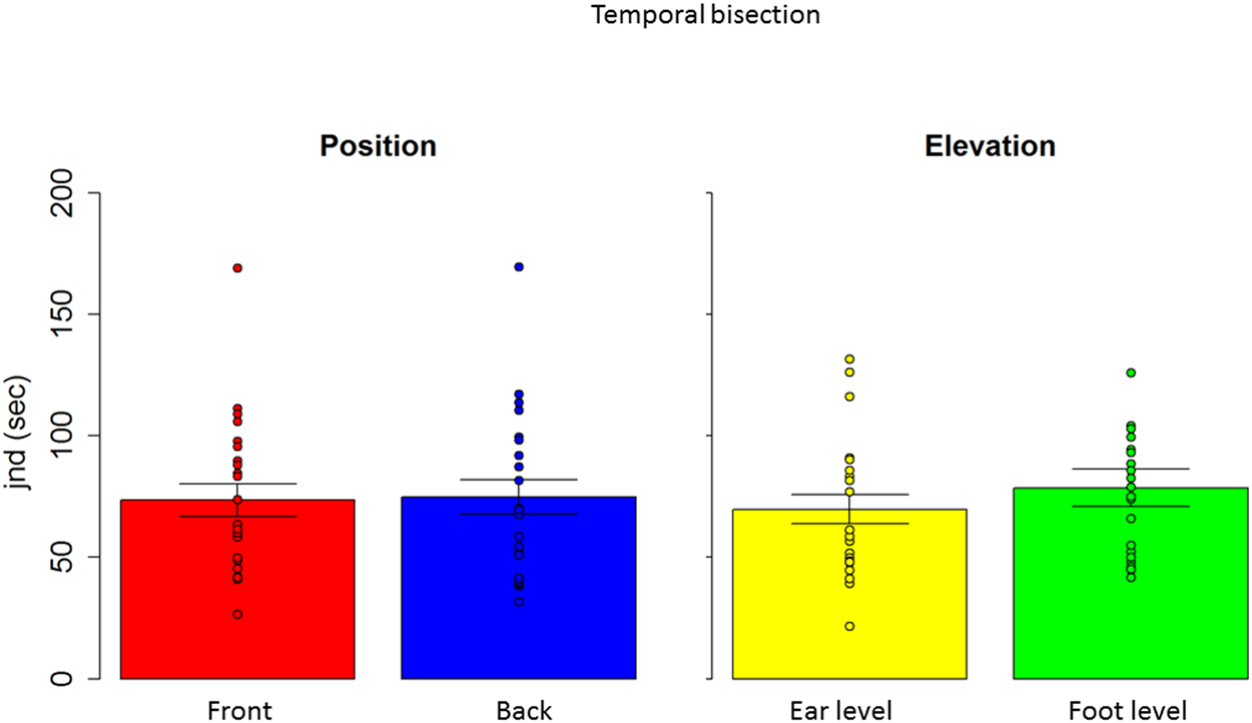
Thresholds in the temporal bisection. No difference between front and back conditions was found at either ear or foot level. Dots represent the performance of each subject.

To find out whether the difference in precision (threshold) at ear and foot level was similar, in a second analysis, we normalized the data by comparing the different degrees sustained by the device at ear and at foot level. Specifically, each angle was divided by the angle corresponding to the span between the first and the last speaker of the relative elevation (i.e. angles at foot level were divided by 19.8°, while angles at ear level by 47.3°). Results are reported in Fig. 6. We performed three independent repeated measure anovas considering sound level (ear vs. foot level) and sound position (front vs. back). Significant results were analyzed by a paired two tailed t-test and we considered P < 0.05 as significant, after applying Bonferroni correction for multiple comparisons. Anova on the spatial bisection showed a significant effect of sound position (F_(1,22)_ = 34, P < 0.01, generalized eta squared (ges) = 0.15). As can be seen in the top left of Fig. 6, higher precision (lower threshold) (t_(22)_ = −5, P < 0.01) was found in the frontal (red bar) space (0.09 +/− 0.01), compared to the back (blue) space (0.14 +/− 0.01). While no differences were found between sound level (F_(1,22)_ = 0.38, P = 0.5, ges = 0.005), as shown in the bottom left of Fig. 6, where performance at ear (yellow) and foot (green) level were practically the same, excluding sound distortion due to the floor. Interestingly, the opposite pattern emerged in the MAA, where similar levels of accuracy were reported between frontal and rear space (P = 0.1). On the other hand, different precision (threshold) was found between ear and foot level (F_(1,22)_ = 21, P < 0.01, ges = 0.16). This result is clearly shown at the bottom right of Fig. 6: as can be seen, precision (threshold) at ear (yellow bar) and foot (green bar) level is significantly different (t_(22)_ = −4, P < 0.01), showing higher precision (lower threshold) for the task performed at ear level (0.07 +/− 0.01) compared with foot level (0.13 +/− 0.01). Significantly, no differences between factors were found in the temporal bisection threshold (P > 0.05), as reported in Fig. 7, suggesting that the complexity of performing the bisection task was similar for the 4 spaces. These results strongly suggest that the auditory metric of sounds coming from the front and back space is differently mapped in space, and that MAA and spatial bisection tasks rely on different cognitive mechanisms.

## Discussion

The aim of this study was to better understand the role of vision in the development of auditory spatial representation. An extended piece of literature reports that stimuli coming from different portions of space surrounding our body (left vs. right, far vs. near) are processed by our brain in different ways. However, only few studies have directly compared frontal and rear space^11–13,53^. Back space is still less investigated, despite it offering the possibility to understand the role of vision in the development of spatial and temporal representation, since in this space also sighted people are naturally blind. In this work, we hypothesized that if vision is needed to calibrate hearing for metric representation, subjects should perform better in the frontal space than in the back space in a spatial bisection task, for which blind individuals show specific impairments^44^. To this end, three tasks were performed by sighted blindfolded individuals: the bisection spatial task, the bisection temporal task and the MAA task. Each task was replicated in the frontal and back space, at two different elevations: ear and foot level. The results confirm our hypothesis, showing that vision is necessary to achieve a good spatial representation during the spatial bisection task. Indeed, during the spatial bisection task, the spatial perception of sounds delivered in the back space was less precise than in the frontal space. Our result is in agreement with previous studies showing a deficit in space representation in blind, but high precision (low threshold) in sighted people^44^. Importantly, no difference in precision (threshold) was reported in the frontal and back spaces for the MAA, as between blind and sighted people^44^ and the temporal bisection task, suggesting that the difference was not due to a different auditory acuity in the four spaces (MAA) and different ability to perform the task per se (temporal bisection task). This finding adds new evidence supporting the necessity of vision for building an auditory spatial metric, thus supporting the idea that visual experience/training is necessary to be able to correctly represent some features of the auditory space. The fact that no differences were present at foot level supports the idea that the effect is not only related to the space around the ear, but it is extended to all space were vision is naturally available, providing more evidence that the effect is due to the role of vision. Moreover, our experiment shows that the influence of vision is space dependent and it is not shared/available in non-visual space. From this evidence, it is possible to speculate that frontal and rear space representations rely on different mechanisms. Previous works have clearly shown that space around us is split into several regions, differently coded by our brain^1,5^. Here we demonstrate that front and back spaces are differently processed, at least in representing auditory spatial metric. Moreover, we show that what makes the difference in this processing is the availability of vision and maybe movement. Indeed, the visual calibration is confined to space where vision and movement can naturally act and it is not shared among spaces; in other words, the possibility to see and act could improve spatial representation resolution of the frontal space compared with the back. Our results also suggest that this calibration occurs only for tasks that require the building of an auditory metric, confirming previous results regarding blind individuals^44^. Significantly, no differences between ear and foot level were found, showing that elevation does not make the task more difficult to perform. As discussed above, similar performance in frontal and rear space was found in the MAA, suggesting that the difference found in the bisection task was not linked to different audio precision (threshold) in these zones, but to a different spatial metric representation. However, significantly higher precision (lower threshold) was reported when the task was performed at ear level, suggesting that the two tasks rely on different mechanisms. Indeed, the spatial bisection task was greatly affected by the longitudinal space, but not by the vertical dimension, in which it was performed, while the MAA presents the exact opposite pattern: it was affected by the vertical dimension, while it was unaffected by longitudinal dimension. These data could be discussed considering that the bisection task needs an allocentric reference frame that, in turn, requires vision, as demonstrated by the study on blind individuals^54,55^. An allocentric reference frame is independent from body position and conformation, as it requires external reference points. To perform the spatial bisection task, subjects have to judge the position of an unpredictable sound with two fixed sounds that are used as external reference points; so the references used to complete the task were always the same in the four spaces considered. The main issue to solve this task, in the four spaces, was the presence of vision and movement. A possible explanation is, therefore, that the lack of visual and motor experience in the back space leads to poor auditory calibration that, in turn, makes it more difficult to use an allocentric reference frame. On the other hand, the minimum audible angle requires the subject to judge the position of a sound with respect to another sound that was always centered on the body (ear or foot). This task could rely more on egocentric (based on the ear or on the foot) reference frame, where vision is less important and can be developed also without visual calibration. This result is supported by results regarding blind individuals, who can perform this task, despite being impaired in the spatial bisection task. The results on blind people^44^ lead to think that the principal role of hearing calibration is provide by vision (as they can move normally). However, in the backspace movement is not possible, so we cannot exclude its role in building a spatial representation. Some works showed that there are no differences in relearning changed spectral cues for frontal or back locations, suggesting that vision is not essential in recalibrating the auditory spatial representation. This is true for simple spatial tasks as sound source localization^27,28^, minimum audible angle^44^, but not in complex task^44^, where an allocentric reference frame is necessary. Taken together, these studies suggest that vision is important to develop more complex auditory spatial skills, as the formation of auditory metric. Our results are in line with this view; indeed people performed similarly well the MAA in the frontal and in the back space, while they were impaired in the bisection task only in the back space. These results are in agreement with previous study^44^ showing that blind people were impaired in the bisection task but not in the MAA. Finally we found dissociated difference in the two spatial tasks (i.e. ear vs foot in MAA, while front vs back in bisection task), but not in the temporal task. This results suggest that the differences between spaces are due to different spatial representation more than to physical parameters of the sound (i.e. ITD, ILD) that were constant across tasks

The results of the temporal bisection task are crucial. The absence of any statistical difference between factors (subjects performed equally in the four spaces) confirmed that the task per se was similarly performed in terms of attentional resources and difficulty. Our results fit well with the literature about space perception and visual calibration, confirming that vision plays an important role in the representation of auditory frontal space^56–58^. Regarding the MAA, we found a different degree threshold then what reported in Mills^59^. This discrepancy could be explained considering differences between the procedures and setups used in the two experiments (e.g. sinusoid vs pink noise sound, different distances/vertical coordinate of sound sources, anechoic vs normal experimental room). Importantly, the present results on the MAA parameter are in agreement with Gori *et al*.^44^ that used a set up and procedure similar to those adopted in our experiment. Here, we showed for the first time that a lack of vision in the back (blind) zone of our body can impact on the development of specific auditory spatial skills, which are required to solve complex auditory spatial tasks. This also suggests that the lack of vision in invisible space leads to the same impairment, although in a less serious form, as that observed in blindness^44^. In conclusion, this study supports the concept that vision and, maybe movement is necessary to calibrate hearing in spatial representation, showing a difference in the performance of auditory tasks in the frontal and rear space. Moreover, our results provide a new insight about the characteristics of the multiple spaces around us, suggesting that different senses may play different roles in calibrating different spaces.
